# Rehabilitation of Individuals With Diabetes Mellitus: Focus on Diabetic Myopathy

**DOI:** 10.3389/fendo.2022.869921

**Published:** 2022-04-14

**Authors:** Daniela Bassi-Dibai, Aldair Darlan Santos-de-Araújo, Almir Vieira Dibai-Filho, Lisiane Fernanda Simeão de Azevedo, Cássia da Luz Goulart, Gabriela Costa Pontes Luz, Patrick Rademaker Burke, Adriana Sanches Garcia-Araújo, Audrey Borghi-Silva

**Affiliations:** ^1^ Postgraduate Program in Environment, Universidade Ceuma, São Luís, Brazil; ^2^ Postgraduate Program in Physical Therapy, Universidade Federal de São Carlos, São Carlos, Brazil; ^3^ Postgraduate Program in Adult Health, Universidade Federal do Maranhão, São Luís, Brazil; ^4^ Department of Physical Therapy, Universidade Ceuma, São Luís, Brazil; ^5^ Department of Medicine, Universidade Federal do Maranhão, Pinheiro, Brazil; ^6^ Department of Physical Therapy, Universidade Federal de São Carlos, São Carlos, Brazil

**Keywords:** diabetes mellitus, diabetic myopathy, cardiometabolic rehabilitation, physical exercise, muscular strength

## Abstract

Diabetes mellitus (DM) is a chronic metabolic disease characterized by high blood glucose levels, causing serious damage to the cardiovascular, respiratory, renal and other systems. The prevalence of type 2 diabetes mellitus (T2DM) was 6.28% in 2017, considering all age groups worldwide (prevalence rate of 6,059 cases per 100,000), and its global prevalence is projected to increase to 7,079 cases per 100,000 by 2030. Furthermore, these individuals are often affected by diabetic myopathy, which is the failure to preserve muscle mass and function in the course of DM. This happens in type 1 diabetes mellitus (T1DM) and T2DM. As skeletal muscle plays a key role in locomotion and glucose homeostasis, diabetic myopathy may contribute to additional complications of the disease. In addition, chronic hyperglycemia is associated with lung functional changes seen in patients with DM, such as reduced lung volumes and compliance, inspiratory muscle strength, and lung elastic recoil. Thus, the weakness of the inspiratory muscles, a consequence of diabetic myopathy, can influence exercise tolerance. Thus, moderate strength training in T2DM can contribute to the gain of peripheral muscle strength. Although the literature is robust on the loss of mass and consequent muscle weakness in diabetic myopathy, triggering pathophysiological factors, the impact on functional capacity, as well as the prescription of physical exercise for this condition deserves to be further explored. This review aims to explore the consequences of diabetic myopathy and its implication in rehabilitation from prescription to safety in the practice of physical exercises for these individuals.

## Introduction

### Muscle as a Key Structure in Diabetes Mellitus: Diabetic Myopathy

Diabetic myopathy is a disease that affects individuals with type 1 (T1DM) or type 2 diabetes mellitus (T2DM). Diabetes mellitus (DM) is associated with reduced mitochondrial function, including decreased mitochondrial number (1), impaired lipid oxidation (2, 3), and excessive production of reactive oxygen species (ROS) (4–6). This scenario of mitochondrial imbalance induces atrophy, transition from oxidative to glycolytic fiber type (7, 8), resulting in skeletal muscle changes, such as muscle weakness, consequent exercise intolerance and decreased quality of life (9, 10).

Glucose uptake in skeletal muscle can occur through several signaling pathways that lead to translocation of the glucose transporter 4 (GLUT4). Thus, there are separate pathways for exercise- and insulin-stimulated glucose uptake. However, the individuals with DM have glucose uptake disorders in peripheral tissues (11).

There are numerous signaling pathways that lead to the intracellular translocation of GLUT4 to the surface of the muscle cell, being glucose-dependent pathways such as insulin receptor substrate-1 (IRS1) and phosphatidylinositol 3-kinase (PI 3-kinase), which then require intracellular insulin signaling to initiate its binding to a specific membrane receptor mentioned above (12). On the other hand, there are insulin-independent signaling pathways, which are activated through muscle contractions, such as adenosine monophosphate-activated protein kinase (AMPK), nitric oxide (NO), bradykinin, AKT, ROS and calcium (13).

In view of the above, it is known that individuals with T2DM have reduced insulin-stimulated glucose uptake in skeletal muscle, since they have insulin resistance. However, exercise-stimulated glucose uptake is maintained during resistance to insulin, as it does not depend on the AKT signaling pathway of the GLUT4 translocation (11).

### Impact of DM on Exercise Capacity

Many are the mechanisms that can lead to a decrease in exercise capacity in this population, with different degrees of functional impairment in the most diverse systems that cooperate in the delivery and use of oxygen were observed, because the supply, demand and consumption of this energy substrate are the result of an interaction of multiple physiological functions, such as pulmonary, cardiovascular and musculoskeletal functions (aerobic capacity, force generation and perception of fatigue) (14).

A recent published review brings with it an integrated approach to physiology in light of the limitations observed in this population that culminate in reduced exercise capacity. Based on cardiopulmonary exercise test (CPET) results, the authors elucidate four pathophysiological determinants that may explain exercise limitation, namely: a) cardiogenic (inadequate adjustment of cardiac output); b) skeletal myogenic (reduced ability to generate force and early fatigue); c) vasogenic (insufficient perfusion and oxygenation); d) neurogenic (impaired cardiopulmonary neural control during exercise) (14).

Therapy for most chronic diseases involves physical training to slow or reverse disease progression (15). During physical exercise, hormones such as insulin and glucagon are responsible for controlling glucose absorption, and the balance between insulin and regulatory hormones may vary based on the type, intensity and duration of exercise (16).

In individuals with T1DM, in the face of aerobic exercise, there is a failure in the circulating levels of insulin, limiting the production of glucose by the liver and facilitating an increase in the elimination of glucose in the skeletal muscle. Contrastingly, during anaerobic exercise, an increase in catecholamines and a failure in circulating levels of insulin at the end of vigorous exercise in individuals with T1DM increases glucose production by the liver while limiting glucose elimination from skeletal muscle, as well, due to this imbalance in glucose production and utilization, circulating glucose levels increase and hyperglycemia may occur (16, 17).

The aerobic exercise promotes improvement in glycemic control observed by fasting glucose and glycated hemoglobin (HbA1c) when compared to control groups (18, 19). On the other hand, a systematic review and meta-analysis showed that aerobic training does not alter HbA1c levels in T1DM (20), although there is an improvement in insulin sensitivity and reduced exogenous insulin requirements in this population (21).

Cuff et al. (22), studying women with T2DM, report that the increase in muscle mass achieved by resistance training contributes to glucose uptake without altering the intrinsic ability of the muscle to respond to insulin, while aerobic exercise improves its absorption capacity through greater insulin action, independently of changes in muscle mass. That is, the type of exercise seems to act differently on muscle glucose metabolism. This fact can be explained by studies that showed that resistance exercise can significantly increase the strength of the skeletal muscles and consequently the cross-sectional area of the muscles, resulting in an increase in the number of insulin receptors and an improvement in insulin sensitivity (23, 24).

Furthermore, previous studies have shown that concurrent exercise, that is, aerobic and resistance exercise in the same section, in patients with DM, lead to an improvement in HbA1c as well as an increase in muscle strength (25, 26).

### Exercise Improves Mitochondrial Function in Diabetic Myopathy Muscles

Moderate levels of ROS are required for normal muscle strength production; however, excess ROS can lead to muscle fatigue and contractile dysfunction (27). Major endogenous sources of ROS in skeletal muscle include mitochondria, nicotinamide adenine dinucleotide phosphate hydrogen oxidase (NOX) and xanthine oxidase (XO) (28). Under physiological conditions, ROS are released as by-products of cellular respiration by mitochondria. Thus, mitochondria-derived superoxide anion radical can be observed in both resting and exercising muscle (29).

However, the physical training has been used as an important component in improving insulin sensitivity and muscle glucose uptake, capable of favoring the translocation of the GLUT4, in addition to contributing to the increase in muscle mass and, consequently, increase carbohydrate metabolism and prevent the deleterious effects of myopathy (30). Additionally, the regulation and maintenance of the mitochondrial network constitute mechanisms important underlying factors to improve metabolic capacity, which is one of the most important skeletal muscle adaptations, tissue with high expression of mitochondria and strongly dependent on oxidative phosphorylation for energy formation (31, 32).

Although it is known that the mitochondria of individuals with DM, especially T2DM, are morphologically smaller, quantitatively less numerous and with a lower oxidative capacity, physical exercise can contribute to the restoration of the content and functionality of these organelles (33, 34). The mechanism that justifies these benefits is associated with the fact that physical exercise contributes, in a comprehensive way, to the activation of the peroxisome proliferator-activated gamma coactivator (PGC-1α), a transcriptional coactivator responsible for regulating genes involved in energy metabolism and mitochondrial biogenesis, responsive to both acute and chronic physical training as the musculature adapts to new metabolic demands (31, 34, 35).

Nevertheless, oxidative stress biomarkers play a relevant role in the pathogenesis of DM and the complications associated with the disease, in addition to being a major contributor to mitochondrial dysfunction (36, 37). In this context, changes in the oxidant and antioxidant balance are associated with high blood glucose concentrations, leading to the production of ROS and lower antioxidant activity (37). The diabetic condition of hyperglycemia leads to the induction of ROS productivity and this productivity is associated with several cellular sources that produce free radicals, especially those involving normal or abnormal metabolism of cellular substrate, such as: non-enzymatic glycation of proteins, electron transport in mitochondria, glucose oxidation and glycation of antioxidant enzymes. Furthermore, it is known that the ketogenic condition, characterized by high levels of ketone bodies, can contribute to ROS formation *via* lipid peroxidation. The subsequent increase in these ROS associated with the hyperglycemic diabetic state leads to decreased insulin gene expression and secretion, deteriorates cellular function, increases insulin resistance, and can ultimately lead to apoptosis (27, 38–41).

Different training modalities have been investigated in this population. However, there is a difficulty in recommending a training intervention for patients with DM that is much superior in terms of effectiveness when it comes to improvement in mitochondrial function, oxidative stress and myopathy, although, still, the literature supports the prescription of combined exercises (aerobic of moderate intensity with resistance training) in view of the benefits achieved in glycemic control and insulin sensitivity, which end up presenting positive results in these deficits (42–44).

Aerobic training has been well documented in terms of qualitative and quantitative improvements in mitochondria, contributing to increased sensitivity to insulin and skeletal muscle mitochondrial protein (42–44). However, it is not surprising that the increase in aerobic capacity resulting from this type of training improves mitochondrial quantity and functionality since this type of training is highly dependent on oxidative phosphorylation and this metabolic pathway occurs essentially in the mitochondria.

Other types of physical training, including strength training and high-intensity interval training (HIIT), have also been reported to be able to increase mitochondrial content and functionality in the skeletal muscle of patients with DM. Particularly, HIIT leads to similar metabolic adaptations compared to traditional resistance training when it comes to improving maximal oxygen uptake and increasing mitochondrial content in healthy individuals. In the diabetic population, this exercise modality still lacks investigations, although, a previous study demonstrated that HIIT can be effective not only in improving glucose metabolism but also resulting in an increase in mitochondrial content in patients with T2DM (45).

### Exercise Attenuates Oxidative Stress and Increases the Antioxidant Capacity in Muscle Against Diabetic Myopathy

Several interrelated mechanisms may explain the increase in ROS and decrease in antioxidant protection in this population. However, the production of ROS cannot always be understood as a villain, since in adequate concentrations they are involved in important physiological processes. Although not fully understood, glycolysis, inactivation of antioxidant enzymes, disturbance in nitric oxide metabolism and intracellular formation of advanced glycation end products are responsible for increased production of free radicals and consequently imbalance between oxidation-reduction (36, 46).

The regular physical exercise continues to be recommended based on strong evidence, however, such benefits offered by the different exercise modalities depend on the training dose. Several studies have proved that although regular physical training leads to antioxidant protection, strenuous exercise can induce an increase in oxidative stress with the activation of metabolism of lipids, proteins and even genetic material (36, 47, 48).

Although skeletal muscle contraction and physical exercise contribute to the development of a stressful oxidative condition in the intracellular and extracellular environment, capable of contributing to the condition of insulin resistance and glucose intolerance; however, exercise-induced ROS are considered important mediators of glucose metabolism, leading to consider this paradox between harms, advantages and their dual role in glucose homeostasis as challenging (49).

Paradoxically, among the mechanisms capable of increasing insulin sensitivity and glucose uptake during and after exercise, the reactions that characterize oxidation-reduction have gained prominence in recent years due to the ability of redox signaling to be essential for optimal physiological functioning. and improved insulin-stimulated glucose uptake (50, 51). In this context, the benefits achieved by exercise are directly linked to the transient increase in the generation of ROS that promotes, over time, with the practice of regular exercise, adaptation and adequate regulation of antioxidant defenses and reduction of systemic oxidative stress markers that, consequently, will culminate in improvements in insulin regulation and glucose metabolism (49).

In fact, in DM, physical exercise reduces the production of ROS and leads to adaptations in the antioxidant capacity, preventing cell damage (52). But how can exercise mitigate oxidative stress and increase the antioxidant capacity of skeletal muscle? The answer to this question is multifactorial and even depends on the type of exercise performed, considering that the formation of ROS depends on the type, intensity, duration and frequency of exercise, as well as the individual antioxidant potential. In skeletal muscle, the amount of ROS produced during exercise, related to the factors previously mentioned in the different types of training, lead to substantial increases in enzymatic and non-enzymatic antioxidant efficiency, especially superoxide dismutase 1 and 2 (SOD1 and SOD2), which can increase its efficiency from 20 to 110%, and glutathione peroxidase (GPX1), which can increase its effectiveness by 180%, in addition to favoring greater mitochondrial functionality in capturing free radicals (52, 53).

### Evaluation and Safety for Exercise Prescription in Diabetic Myopathy

Among the exercise capacity assessment methods, the CPET remains the gold standard for this purpose. The main care for performing CPET in these individuals is the management of hypoglycemia, hyperglycemia and ketosis, and knowledge of the medications used and planning prior to the test are strongly recommended. CPET should not be performed in the presence of ketosis, regardless of glycemic level (54–56).

The aerobic training improves glycemic control in patients with T1DM (57), despite the association between continuous aerobic exercise and hypoglycemia (58). On the other hand, the literature is robust in demonstrating that exercise sessions composed of strength training exercises or HITT reduce the risk of hypoglycemia during and after physical exertion, when compared to aerobic exercises performed in a continuous in insulin-dependent patients (17, 59). In this scenario, a recent consensus on T1DM strongly recommends the practice of anaerobic exercises for these patients, since this type of physical demand is associated with smaller declines in blood glucose or even increases in glucose levels (60). Thus, anaerobic exercises seem to be safe, especially in those who use insulin.

Therefore, recently published literature aiming to provide general considerations and new directions in the management of T1DM suggests that a training program composed of a combination of HIIT and strength exercise can minimize the rapid drop in blood glucose associated with exercise in T1DM. They further speculate that this is due to increased hepatic glucose production stimulated by catecholamine release and an increased transient inhibition of insulin-mediated glucose uptake due to lactate production (61).

Although CPET is the gold standard for assessing exercise capacity and prescribing safe exercise, unfortunately, a minority of centers have such a resource, as it is an expensive test and requires a large number of trained professionals. Given this, some safe and viable assessment tools can be used, mainly in T2DM, such as the six-minute walk test and the step test. As important as aerobic exercise is strength training, which can be based on 1-repetition maximum assessment, as demonstrated by studies with individuals with T1DM and T2DM (62–64).

Not least, the autonomic nervous system, which is significantly impaired in individuals with DM, deserves attention when evaluating these patients. Heart rate variability (HRV) is one of the most used tools to assess autonomic control in both T1DM and T2DM individuals. It can be performed using linear measurements in the time and frequency domains in addition to non-linear measurements. Other autonomic assessment methods include: heart rate response to the Valsalva maneuver, diastolic blood pressure response to isometric exercise, systolic blood pressure response to standing, heart rate response to standing and holter.

Finally, a more targeted assessment for the diagnosis of sensorimotor neuropathy can be achieved through nerve conduction velocity studies (65–67).

### Novel Approaches of How to Prevent the Progression of the Disease and Improve Physical Fitness

The benefits of secondary prevention strategies for chronic disease, including T2DM, targeting lifestyle modification and risk factor management have been well established worldwide (68, 69). However, unfortunately, the adherence of these patients considering the lifestyle changes from a more sedentary to an active pattern is still a big challenge (70, 71). Despite the numerous benefits of formal rehabilitation (clinical centers or home-rehabilitation) in improving physical health and quality of life, combined with benefits in glycemic control, it is important to consider that the sessions can be relatively monotonous and unattractive over time. Therefore, other ways of delivering these services must be emphatically promoted by the public and private sector. Therefore, innovative and cost-effective interventions centered on the patients’ preference to enhance adherence in physical training programs are urgently needed.

In this context, programs that include multimodal interventions, consisting of physical exercise, nutritional changes, behavioral therapy and cognitive approaches have shown interesting results in the medium (72) and long term (73, 74). Considering the behavioral changes related to a healthier lifestyle, recent technologies have been incorporated as a way to expand the distribution of new interventional approaches to patients with T2DM. Mobile text messaging interventions can improve glycemic control (75, 76) and improve active behavior (77) of these patients. Technology-assisted can provide self-monitoring, and it was concluded that the technology-assisted self-monitoring approach was beneficial, safe, and feasible to use for positive lifestyle change.

**Figure 1:**
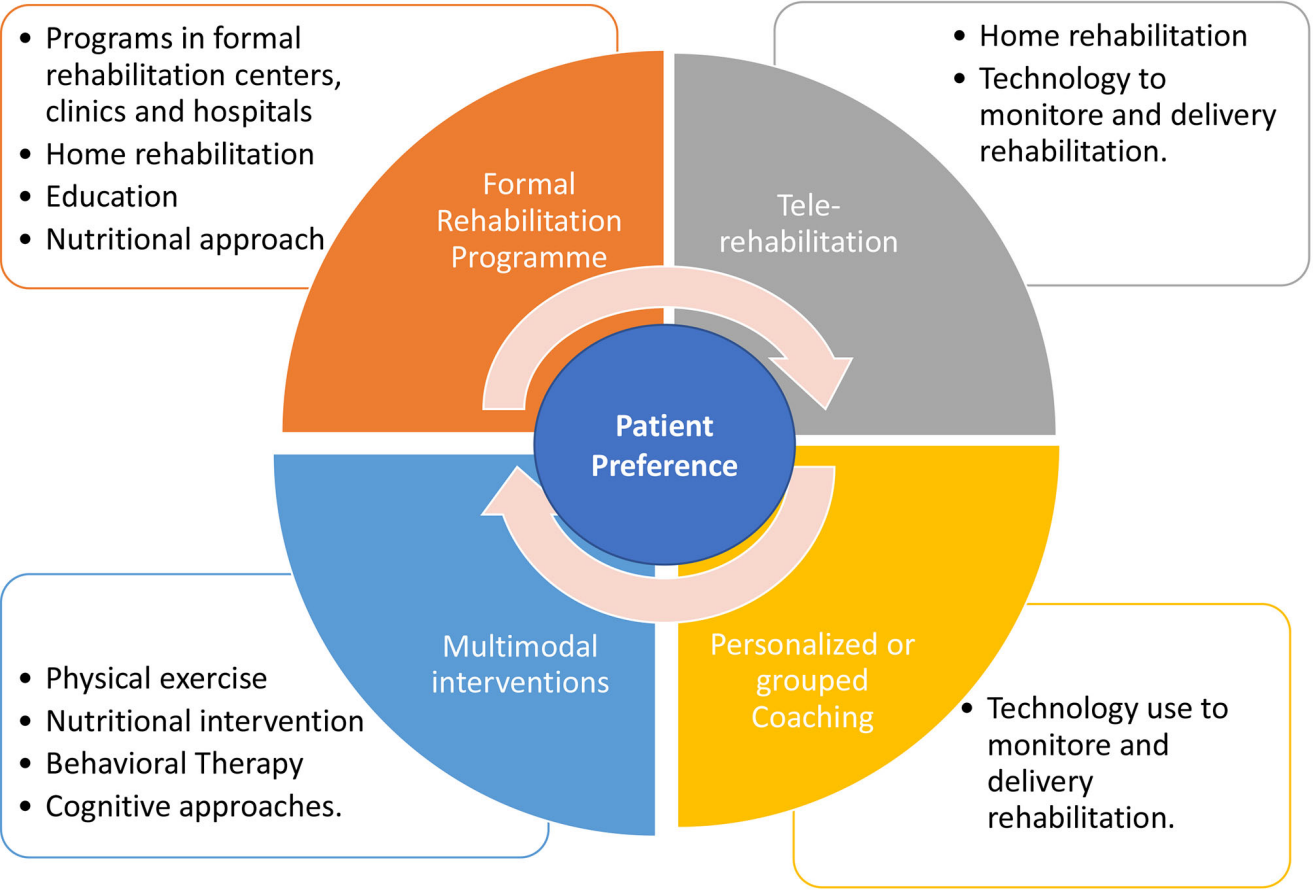
Summary of components of rehabilitation program on the population with T2DM considering the patient preference.

Apps and text messages can help patients in monitoring, managing and expanding their education in disease control. Furthermore, such technologies have proved to be interesting participant engagement strategies, adjusting to the routine of many patients (78, 79). In addition, personalized coaching, goal setting, peer support groups, use of technology as physical activity monitors were proven to increase the level of physical activity for large patient populations (80). **Figure 1** summarizes the components of rehabilitation program on the population with T2DM considering the patient preference.

## Discussion

Patients with diabetic myopathy are unable to exercise and, consequently, perform their activities of daily living in an even more impaired way. However, the literature remains very heterogeneous and these mechanisms need further clarification in view of the divergences found in the scientific literature that address the theme (14).

In this context, cardiopulmonary rehabilitation involving a combined exercise program can improve antioxidant capacity and attenuate oxidative stress and functional improvements (52), and, attenuating the pathophysiological mechanisms of diabetic myopathy proving to be safe and effective in improving exercise capacity and consequently functionality (25), being a cornerstone of non-pharmacological therapeutic strategies in DM. Finally, rehabilitation programs can improve the prognosis of mortality for all the causes that, in this population, is notably high (14, 67, 81).

There are many studies addressing diabetic neuropathies but few focusing on diabetic myopathy. Based on the limited number of studies to date, it is evident that further studies on the effects of physical exercise in the treatment and progression of diabetic myopathy be carried out with an adequate sample of patients. Besides that, controlled, randomized and double-blind studies, are essential to clarify the main protective effects that physical exercise can develop in the body of individuals who are affected by the disease.

In addition, studies assessing interventions to improve the muscle mass and strength in diabetic patients, before diabetic myopathy development, should be encouraged, as well as studies addressing to investigate the PGC-1 α function in the diabetic myopathy during the rehabilitation’s programs utilizing the exercise.

## Author Contributions

DBD, ADSA, ADVF, LFSA, CLG, GCPL, PRB, ASG-A, and ABS designed, wrote and revised this manuscript. All authors contributed to the article and approved the submitted version.

## Funding

This work as partially supported by Coordenação de Aperfeiçoamento de Pessoal de Nível Superior (CAPES, finance code 001).

## Conflict of Interest

The authors declare that the research was conducted in the absence of any commercial or financial relationships that could be construed as a potential conflict of interest.

## Publisher’s Note

All claims expressed in this article are solely those of the authors and do not necessarily represent those of their affiliated organizations, or those of the publisher, the editors and the reviewers. Any product that may be evaluated in this article, or claim that may be made by its manufacturer, is not guaranteed or endorsed by the publisher.
